# A high anti-inflammatory response is associated with intermediate-term mortality in patients with sepsis

**DOI:** 10.1186/2197-425X-3-S1-A79

**Published:** 2015-10-01

**Authors:** JF Frencken, LA van Vught, DS Ong, PMC Klein Klouwenberg, J Horn, MJM Bonten, T van der Poll, OL Cremer

**Affiliations:** Julius Center, University Medical Center Utrecht, Utrecht, Netherlands; Center for Experimental and Molecular Medicine, Academic Medical Center, University of Amsterdam, Amsterdam, Netherlands; Center for Infection and Immunity, Academic Medical Center, Amsterdam, Netherlands; Medical Microbiology, University Medical Center Utrecht, Utrecht, Netherlands; Intensive Care, Academic Medical Center, University of Amsterdam, Amsterdam, Netherlands; Division of Infectious Diseases, Academic Medical Center, University of Amsterdam, Amsterdam, Netherlands; Intensive Care, University Medical Center Utrecht, Utrecht, Netherlands

## Introduction

Sepsis is characterized by a complex systemic inflammatory response to infection. While an overwhelming pro-inflammatory response is held responsible for early deaths, subsequent anti-inflammatory cytokine production may lead to immunosuppression and secondary infections. This has been suggested as a cause of intermediate-term deaths[1].

## Objectives

To study the relation between a persistent anti-inflammatory response and day 4-14 mortality in patients with severe sepsis and septic shock who have survived beyond the initial pro-inflammatory phase.

## Methods

We included consecutive patients admitted with severe sepsis or septic shock to the Intensive Care Units (ICU) of 2 tertiary care centres in The Netherlands between January 2011 and July 2013 with a length of stay of at least 4 days. We excluded patients with prior immune deficiency. The anti-inflammatory response was assessed through interleukin (IL)-10 plasma concentrations on admission, day 2, and day 4 using BD™ CBA Flex Set system immunoassays. We categorized patients into 3 groups (low, moderate, high) based on day 4 IL-10 percentiles ( < 25^th^, 25-75^th^, >75^th^) and change from day 2 values. We measured IL-6 as marker of pro-inflammation. We used multivariable logistic regression analysis to study the relation with day 4-14 mortality and control for confounding.

## Results

We enrolled 485 patients; of these, we excluded 116 cases because of known immune deficiency and 19 cases because of missing plasma samples, leaving 350 subjects for analysis. A total of 148 (42%) patients were categorized as having a low anti-inflammatory response, 122 (35%) as moderate, and 80 (23%) as high. The groups were similar with respect to age, gender, and ICU length of stay (LOS), but patients with high anti-inflammatory response had higher Apache IV scores and were diagnosed with more abdominal and less pulmonary infections. Mortality between day 4 and 14 was 14%, 9% and 36% for patients with low, moderate, and high IL-10 levels, respectively (p= < 0.01). After adjustment for age, comorbidities, sequential organ failure assessment score, site of infection and IL-6 response in the first 4 days of ICU admission, a persistent high anti-inflammatory response on day 4 remained independently associated with increased mortality (crude odds ratio high vs. low 3.4; adjusted 2.8, 95% CI 1.4-5.7).

## Conclusions

High anti-inflammatory response after 4 days is an independent risk factor for intermediate-term mortality in critically ill patients with sepsis.

## Grant Acknowledgment

This study was performed within the Molecular diagnosis And Risk stratification of Sepsis (MARS) project (grant-04l-201).

**Table 1:** Patient characteristics.

	Low IL-10 level (n = 148)	Moderate IL-10 level (n = 122)	High IL-10 level (n = 80)	p-value
Age, median (IQR)	65 (56-73)	62 (53-73)	66 (59-73)	0.22
Gender (male), n (%)	94 (64%)	75 (62%)	45 (56%)	0.55
Charlson Comorbidity Index, median (IQR)	4 (0-10)	4 (0-9)	5 (0-11)	0.75
Apache IV score, median (IQR)	82 (67-99)	83 (69-102)	87 (71-113)	0.06
ICU length of stay, median (IQR)	10 (6-17)	10 (7-19)	10 (7-19)	0.38
Total SOFA score day 3, median (IQR)	8 (6-10)	9 (7-11)	10 (8-13)	< 0.01
IL-6 day 4 (pg/ml)*, median (IQR)	3 (2.3-4)	3.9 (3.0-4.7)	4.7 (4-5.5)	< 0.01
Day-14 mortality, n (%)	21 (14%)	11 (9%)	29 (36%)	< 0.01

IQR=Interquartile range. SOFA = Sequential Organ Failure Assessment. Difference between groups (low, moderate, high) was tested by one-way ANOVA or Kruskal-Wallis test for continuous variables as appropriate. Differences between groups for categorical variables was tested for using a Chi-square test. * Log transformed

## References

[1] Hotchkiss RS, Monneret G, Payen D (2013). Sepsis-induced immunosuppression: from cellular dysfunctions to immunotherapy. Nat Rev Immunol.

